# Calcitriol-Mediated Hypercalcemia Due to Liver Metastases in a Patient With Primary Pancreatic Neuroendocrine Tumor

**DOI:** 10.1210/jcemcr/luae209

**Published:** 2024-11-14

**Authors:** Katherine I Wolf, Oxana V Crysler, Robert Fontana, Sima Saberi

**Affiliations:** Department of Internal Medicine, Division of Metabolism, Endocrinology & Diabetes, Michigan Medicine, Ann Arbor, MI 48109, USA; Department of Internal Medicine, Division of Hematology and Oncology, Michigan Medicine, Ann Arbor, MI 48109, USA; Department of Internal Medicine, Division of Gastroenterology and Hepatology, Michigan Medicine, Ann Arbor, MI 48109, USA; Department of Internal Medicine, Division of Metabolism, Endocrinology & Diabetes, Michigan Medicine, Ann Arbor, MI 48109, USA

**Keywords:** hypercalcemia, 1,25-dihydroxyvitamin D, metastatic pancreatic neuroendocrine tumor

## Abstract

Hypercalcemia is most commonly associated with primary hyperparathyroidism or malignancy in the setting of elevated parathyroid hormone-related protein or bone metastases. Calcitriol (1,25-dihydroxyvitamin D)-mediated hypercalcemia is rare and typically associated with granulomatous conditions; however, other solid-organ etiologies have been reported. Here, we detail the case of a 62-year-old man with metastatic pancreatic neuroendocrine tumor (pNET) with hypercalcemia refractory to traditional bisphosphonate therapy in the setting of vastly elevated 1,25-dihydroxyvitamin D. Only after initiation of chemotherapy with capecitabine and temozolomide did his serum calcium consistently improve and 1,25-dihydroxyvitamin D begin to decrease. There are fewer than 5 reported cases of a pNET resulting in calcitriol-mediated hypercalcemia. Prompt initiation of treatment for the underlying condition can result in a significant improvement in serum calcium or 1,25-dihydroxyvitamin D. Multiple reports have also demonstrated success with high-dose steroid administration in patients with other solid-organ etiologies of calcitriol-mediated hypercalcemia, but this has not yet been reviewed in the pNET population.

## Introduction

In 90% of cases, hypercalcemia is a result of primary hyperparathyroidism or malignancy [1]. Hypercalcemia of malignancy is predominately related to excess parathyroid hormone-related peptide (PTHrP) or the presence of metastatic bone disease [2]. However, 1,25-dihydroxyvitamin D (calcitriol) is a rare, but known etiology of hypercalcemia, particularly with granulomatous conditions. In 2013, a systemic review from Australia identified 101 patients with calcitriol-mediated hypercalcemia, finding that sarcoidosis (49%), hematologic malignancies, mainly lymphoma (17%), and mycobacterium infections (8%) were the most frequent etiologies of calcitriol-mediated hypercalcemia [3]. At the time, there were 5 reported cases of solid-organ malignancy related disease as a result of ovarian clear cell cystadenocarcinoma, seminoma, metastatic squamous cell carcinoma of tongue, metastatic adenocarcinoma of unknown primary, and non-small cell lung carcinoma [3].

Over the last decade, multiple cases have highlighted immunotherapy-related [4] or solid-organ tumors resulting in calcitriol-mediated hypercalcemia, including, from an endocrine perspective, gastrointestinal stromal tumors [2] and metastatic pancreatic neuroendocrine tumors (pNET) [5  6  7]. Here, we report a case of metastatic pNET resulting in hypercalcemia secondary to the secretion of 1,25-dihydroxyvitamin D, which improved following the initiation of chemotherapy for the underlying disease.

## Case Presentation

A 51-year-old male individual with prediabetes and depression presented with constipation and abdominal discomfort in the setting of a palpable abdominal mass and was ultimately found to have a 7.9-cm nonfunctional pNET. Resection was attempted, but ultimately aborted, due to tumor adherence to the superior mesenteric artery and aorta. Following a 5-month course of chemotherapy, he underwent en bloc resection with distal pancreatectomy, partial gastrectomy, and partial colectomy without complication. Pathology confirmed a well-differentiated grade 1 pNET with lymphovascular and perineural invasion, 0/25 positive lymph nodes, and negative margins. Over the next several years, he underwent routine surveillance of known liver lesions, but he was not on any treatment. After several stable scans, he ceased surveillance.

Ten years following his initial diagnosis, he presented to the emergency department with right flank pain where computed tomography demonstrated bilateral nephrolithiasis with a 4-mm stone in the proximal right ureter and multiple liver masses measuring upwards of 6.1 × 5.4 × 6.5 cm in the right inferior pole, suggesting neoplasm. Biochemical evaluation demonstrated leukocytosis, anemia, and hypercalcemia to 12.3 mg/dL (3.07 mmol/L) (reference range [RR] 8.2-10.2 mg/dL; 2.2—2.6 mmol/L) without other electrolyte abnormalities or acute kidney injury.

Given that he was afebrile and hemodynamically stable, he was discharged following intravenous fluid administration with prescriptions for cephalexin, hydrocodone-acetaminophen, ketorolac, tamsulosin, and ondansetron with instructions to follow closely with his primary care provider and oncologist.

## Diagnostic Assessment

Over the following weeks, laboratory testing demonstrated continued hypercalcemia above 12 mg/dL (> 3.07 mmol/L) with depressed parathyroid hormone 8 pg/mL (8 ng/L) (RR 15-65 pg/mL; 15-65 ng/L), mildly elevated PTHrP 24 pg/mL (2.4 pmol/L) (RR 11-20 pg/mL; < 2.0 pmol/L), and 1,25-dihydroxyvitamin D elevated above the upper reference limit of detection of 200 pg/mL (> 499 pmol/L) (RR 18-64 pg/mL; 24-45 pmol/L) in the setting of normal levels of 25-hydroxyvitamin D (25 ng/mL; 62.4 nmol/L) (RR > 20 ng/mL; 9-200 nmol/L) and albumin (4.7 g/dL (47.0 g/L) (RR 3.2-4.7 g/dL; 32-47 g/L). Liver function tests, 5-hydroxyindoleactic acid, carcinoembryonic antigen, 24-hour urine protein electrophoresis, chromogranin A, vasoactive intestinal peptide, gastrin, pancreatic polypeptide, glucagon, insulin, and proinsulin levels were unremarkable.

Magnetic resonance imaging demonstrated 9 enlarging hepatic masses ranging in size from 2.5 to 7.5 cm, and 64-copper (Cu) DOTATATE positron emission tomography verified multiple liver foci of intense uptake with maximal standardized uptake values of 86.3 consistent with metastatic NET. There was no evidence of metastatic disease within the head, neck, thorax, pelvis, or osseous structures. Nuclear medicine bone scan was without evidence of metastatic bone disease. Ultrasound guided liver biopsy confirmed metastatic, grade 2, well-differentiated NET positive for synaptophysin and chromogranin.

## Treatment

From a malignancy perspective, he was originally initiated on monthly octreotide injections (20 mg) with active surveillance per the recommendation of a multidisciplinary tumor board and consideration of locoregional therapies with interventional radiology or systemic targeted radiotherapy with 177-Lutetium (Lu) DOTATATE or chemotherapy if evidence of disease progression. Regarding his paraneoplastic mediated hypercalcemia, he received 2 doses of pamidronic acid (90 mg) before transitioning to monthly zoledronic acid (4 mg) infusions, of which he has tolerated upwards of 20 doses without complication.

Following a second opinion at our institution, his octreotide dose was increased, and he was initiated on combination chemotherapy with capecitabine and temozolomide (CAPTEM) more than 1 year following his diagnosis of metastatic NET.

## Outcome and Follow-Up

**Figure 1.**
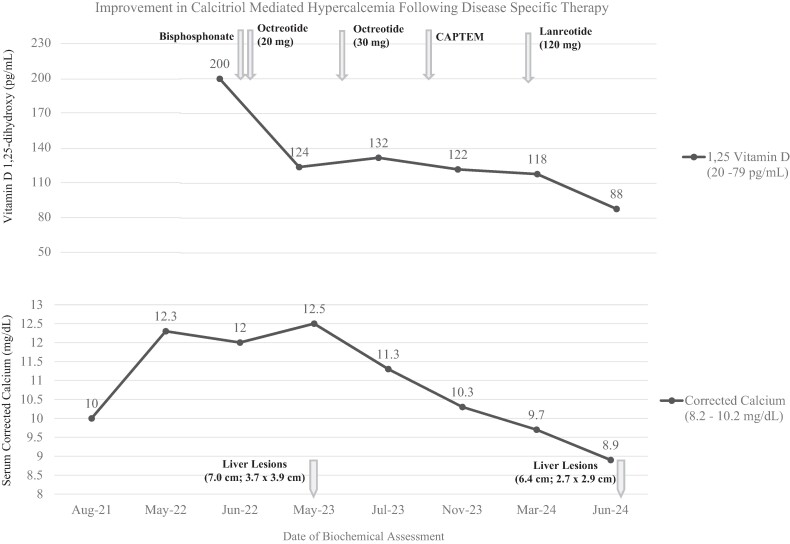
Improvement in calcitriol-mediated hypercalcemia following disease-specific therapy. 1,25-dihydroxyvitamin D levels (TOP) compared to corrected serum calcium levels (BOTTOM) as the patient progressed with therapy, including bisphosphonate, octreotide/lanreotide, and chemotherapy used along the top and size of liver lesions along the bottom. Reference range for 1,25-dihydroxyvitamin D (20-79 pg/mL), corrected serum calcium (8.2-10.2 mg/dL).

After initiating octreotide and bisphosphonate therapy, the patient's calcium and 1,25- dihydroxyvitamin D levels decreased but were still above the upper reference limit of normal (Fig. 1). Within 3 months of CAPTEM therapy, calcium normalized. 1,25-dihydroxyvitamin D continues to decrease, although remains elevated above the upper reference limit of normal. Magnetic resonance imaging of the abdomen 8 months after initiation of CAPTEM therapy demonstrated a decrease in the size of his liver metastasis. He continues on monthly zoledronic acid infusions, as of now, with a hold parameter of calcium less than 8.2 mg/dL (2.5 mmol/L). Even in the setting of continued chemotherapy, which he is tolerating well except for mild diarrhea and change in taste, he reports improved fatigue and energy levels following eucalcemia.

Ongoing discussions continue to occur regarding definitive therapy, including the potential for liver transplantation, particularly in the setting of no overt extrahepatic disease. However, liver-directed therapy is currently being explored as a break from systemic therapy before initiating a transplant evaluation.

## Discussion

Here, we present a case of calcitriol-mediated hypercalcemia in the setting of metastatic pNET. Despite more than a year of monthly somatostatin analogues and bisphosphonate therapy, he remained hypercalcemic with significantly elevated 1,25-dihydroxyvitamin D levels until he initiated systemic chemotherapy with CAPTEM. After 10 months of treatment, he continues to tolerate chemotherapy with minimal side effects and with continued progressive improvement in 1,25-dihydroxyvitamin D. While temozolomide has been linked to hepatotoxicity, his liver function test levels have not increased [8].

While the etiology of calcitriol-mediated hypercalcemia is well-known within granulomatous diseases, less is known in solid-organ malignancies. In sarcoidosis, tuberculosis, and lymphomas, activated macrophages within granulomas convert 25-hydroxyvitamin D to 1,25-dihydroxyvitamin D, producing abnormally high and dysregulated calcitriol [3]. In non-granulomatous diseases, it is hypothesized that 25-hydroxyvitamin D-1-hydroxylase is expressed in macrophages and lymphocytes, which converts 25-hydroxyvitamin D to 1,25-dihydroxyvitamin D. However, the exact physiologic mechanism is unknown [2].

To our best knowledge, there are fewer than 5 reported cases of metastatic pNET resulting in hypercalcemia from excess 1,25-dihydroxyvitamin D. In the 2019 report from van Lierop et al, they were able to demonstrate expression of CYP27B1 in tumoral tissue mRNA, which encodes the converting enzyme (25-hydroxyvitamin D3 1-alpha-hydroxylase) that converts 25-hydroxyvitamin D3 into the active metabolite. The thousand-fold higher expression of tumoral CYP27B1 compared to control samples of cells known to express 1-alpha-hydroxylase was hypothesized as the etiology of elevated calcitriol levels generated by the pNET [6].

When patients with metastatic NET are unable to undergo complete resection of their disease, management is largely based on symptoms, tumor burden, and the rate of disease progression, with initial treatment options in largely asymptomatic patients ranging from watchful waiting to somatostatin analogues [5]. The case presented here is unusual in that [1] despite significantly elevated 1,25-dihydroxyvitamin D, the patient’s PTHrP level was elevated and [2] his metastatic disease burden was relatively moderate and confined to the liver. With respect to the relationship between 1,25-dihydroxyvitamin D and PTHrP, a 2021 case report from Kim et al illustrated dual mechanisms of hypercalcemia (simultaneously elevated PTHrP and 1,25 dihydroxyvitamin D) and argued that a lack of response to bisphosphonate therapy with normalization of calcium following glucocorticoids effectively indicated that PTHrP was not the sole driver of the patient's hypercalcemia [9]. Therefore, our patient's mildly elevated PTHrP may be a secondary contributor to his hypercalcemia of malignancy. With respect to his burden of disease, despite being stable and overall limited, he developed paraneoplastic hypercalcemia, which was a significant contributor in the progression of his treatment plan to chemotherapy, especially when liver transplantation was called into question and deferred until resolution of hypercalcemia. While treatment for metastatic pNET typically proceeds in the above stepwise fashion, early consideration of systemic chemotherapy should be discussed in patients with calcitriol-mediated hypercalcemia as somatostatin analogues and bisphosphonate therapy may not significantly improve hypercalcemia rapidly. In multiple reported cases of metastatic pNET with calcitriol-mediated hypercalcemia, the patients tolerated CAPTEM with limited side effects, which is in line with several studies documenting minimal grade 3 (thrombocytopenia) and grade 4 adverse events and objective radiographic evidence of improvement in 70% of patients with a median progression-free survival between 14 and 18 months [10  11  12].

In 2020, an international panel of experts published consensus recommendations for a treatment algorithm in pulmonary sarcoidosis, including hypercalcemia. First-line therapy remains corticosteroids, typically 20 to 40 mg prednisone daily with careful consideration and titration in patients with diabetes, psychosis, and osteoporosis [13]. There was also agreement that hydroxychloroquine may be useful in managing calcitriol-mediated hypercalcemia as well. In a *Journal of Clinical Endocrinology & Metabolism* review of more than 100 patients with hypercalcemia mediated by 1,25-dihydroxyvitamin D, Donovan et al found that many patients required higher corticosteroid doses, but that there did not appear to be any benefit with respect to serum calcium normalization at doses greater than 50 mg equivalent of prednisone per day [3]. Furthermore, the study reported that the median time to normalization of serum calcium was a little over a week, which matches other studies, but with a significant right skew, likely in the setting of delayed corticosteroid initiation in favor of intravenous hydration and bisphosphonate therapy [3]. It is important to note that in the pNET population, corticosteroids and/or hydroxychloroquine have not been studied, but could potentially offer benefit, particularly in a situation where the oncologist may not consider that initiation of systemic chemotherapy is warranted. Further studies are needed to determine if prompt initiation of corticosteroids in the metastatic pNET population would curb calcitriol-mediated hypercalcemia and delay or prevent the initiation of chemotherapy in otherwise moderate metastatic disease burden.

## Learning Points

- Patients with hypercalcemia and pancreatic neuroendocrine tumors should be evaluated with serum 1,25-dihydroxyvitamin D to evaluate for calcitriol-mediated disease.
- Main stay of treatment for paraneoplastic hypercalcemia is treatment of the underlying malignancy.
- If treatment is not readily available, bisphosphonates and octreotide can be used as temporizing measures. Other reports of paraneoplastic hypercalcemia refractory to the above treatment regimens also note high-dose prednisone therapy as an alternative therapy.

## Data Availability

Data sharing is not applicable to this article as no datasets were generated or analyzed during the current study.

## Abbreviations

Pnet: pancreatic neuroendocrine tumor
PTHrP: parathyroid hormone-related peptide
RR: reference range
